# Effect of trust in village health workers on the use of facility-based follow-up postnatal care services in two districts in the Lao People’s Democratic Republic

**DOI:** 10.1186/s41182-025-00730-8

**Published:** 2025-04-27

**Authors:** Noudéhouénou Credo Adelphe Ahissou, Manami Uehara, Daisuke Nonaka, Inthanomchanh Vongphoumy, Tiengkham Pongvongsa, Khamtoun Ounlienvongsack, Khampheng Phongluxa, Sengchanh Kounnavong, Jun Kobayashi

**Affiliations:** 1 Department of Global Health, Graduate School of Health Sciences, University of the Ryukyus, Okinawa, Japan; 2 Savannakhet Provincial Health Office, Savannakhet, Lao PDR; 3 Sepone District Health Office, Savannakhet, Lao PDR; 4 Lao Tropical and Public Health Institute, Vientiane, Lao PDR

**Keywords:** Trust, Village health volunteers, Follow-up postnatal care, Lao PDR

## Abstract

**Background:**

Despite high coverage of antenatal care services (89.8%) and facility-based deliveries (79.8%), delayed initiation or lack of follow-up postnatal care (PNC) visits remains a challenge in the Lao People's Democratic Republic (Lao PDR). Follow-up PNC encourages healthy lifestyles and monitoring mothers' and newborns' health to decrease postpartum complications and hospital readmissions. While village health volunteers and workers (VHVs/VHWs) are essential for health promotion in Lao PDR, the extent to which mothers' trust in VHVs/VHWs helps promote better service utilization has not been studied.

**Objectives:**

We investigated the trust levels in VHVs/VHWs among ethnic minority mothers and the influence on the use of facility-based follow-up PNC.

**Methods:**

We utilized cross-sectional data from July and August 2024, collected as a baseline survey for a quasi-experimental study conducted in 35 villages across the Sepone and Vilabuly districts. We compared the respondents' PNC usage and characteristics using chi-square tests and Fisher’s exact tests. Standard binary logistic regression analyses were conducted to estimate the effects of various factors on the utilization of facility-based follow-up PNC. Trust in VHVs/VHWs was a construct variable based on their provision of emotional support, relevant information, adequate discussion time, effective care, and the likelihood of future pregnancy-related care.

**Results:**

The study enrolled 241 mothers (mean age 24 years, SD 5.7), including 110 from Sepone and 131 from Vilabuly. Overall, the follow-up PNC coverage rate was 19.0%, and there was no significant difference between Sepone and Vilabuly, despite variations in access to healthcare and engagement with VHVs/VHWs. High trust in VHVs/VHWs was linked to 12.25 times higher odds of utilizing follow-up PNC than low trust (95% CI 2.2–67.8). In addition, having an older child (9–12 months) and immediate PNC utilization were beneficial for subsequent PNC use. Unexpectedly, contact with VHVs/VHWs during prenatal and/or postnatal periods decreased the odds of utilizing follow-up PNC, with distance to health facilities and adherence to traditional gender norms also having a similar negative effect.

**Conclusions:**

Facility-based follow-up postnatal care was critically low among respondents; however, increasing trust in VHVs/VHWs may foster improvements. Along with increasing contact frequency, offering quality support to mothers through VHVs/VHWs while emphasizing the complementary nature of community-based and facility-based care could be beneficial. Effective interventions may also include enhancing immediate PNC and tackling physical accessibility challenges, as well as restrictive gender norms through male involvement.

## Background

Maternal and child morbidity and mortality during the postnatal period continue to pose a global challenge, particularly in low- and middle-income countries. According to the World Health Organization, in 2022 alone, approximately 2.3 million newborn deaths occurred in the first month of life, with almost two-thirds of maternal deaths occurring within the first week after childbirth [1, 2]. During the postpartum period, many women are vulnerable to postpartum depression and anxiety, infections, cardiovascular conditions, and hemorrhages, increasing the risk of complications and hospital readmission [3, 4]. Similarly, in the first month of life, newborns are susceptible to sudden infant death syndrome, feeding problems, jaundice, and infections, such as sepsis and pneumonia [4].

Postnatal care (PNC) services are crucial interventions during the first 6 weeks after childbirth, and enhance the likelihood of survival for both mothers and newborns. In addition to immediate PNC provided before discharge from the place of delivery to address the urgent health needs of mothers and babies, the World Health Organization (WHO) recommends two follow-up PNC visits for continuous maternal and child care [4]. PNC services monitor the health of mothers and newborns, promote healthy lifestyles and good nutrition, provide psychological care for mothers, detect diseases or complications early, and encourage family support. However, while PNC coverage already lags globally in comparison with antenatal care (ANC) and facility-based delivery, follow-up PNC services remain significantly underused [5, 6].

Lao People’s Democratic Republic (Lao PDR), a landlocked country in Southeast Asia, continues to struggle with poor maternal and child health outcomes. Over the past years, Lao PDR has significantly improved maternal and child health (MCH) indicators, increasing antenatal care coverage from 54.2% to 89.8% and skilled birth attendance from 41.5% to 79.8% between 2011 and 2023 [7, 8]. Nonetheless, the use of follow-up PNC services remains alarmingly low. In 2017, only 47.2% of women accessed any PNC services, and less than 2% received follow-up PNC checks after their initial visit [9–11]. Among the country's initiatives, the 2019 Lao Primary Health Care Policy emphasized the role of community health workers (CHWs), known as village health volunteers (VHVs) and village health workers (VHWs), as essential human resources for reproduction and MCH promotion [12]. With no professional training, VHVs/VHWs are often chosen in communities, where they serve, in support of the health system, either as volunteers without a fixed compensation or against a minimum regular stipend [12]. In MCH, VHVs/VHWs conduct home visits to facilitate educational activities and health monitoring and promote access to essential obstetric services [13–15].

Despite extensive evidence on the effectiveness of CHWs for health promotion, the extent to which communities trust CHWs and how this influences healthcare services remain understudied [16–19]. Furthermore, no study has explicitly explored the effect of trust in CHWs on MCH service utilization in rural communities. Trust in CHWs reflects the strength of relationships with communities and reliance on them, constituting leverage for intervention efforts to promote adherence to health recommendations [20, 21]. Therefore, the present study assesses the extent to which trust in VHVs/VHWs influences the uptake of facility-based follow-up PNC in rural Lao PDR. The findings will help design targeted community-based interventions to increase service utilization and reduce poor maternal and infant health outcomes in underserved areas.

## Methods

### Study design and area

We used cross-sectional data from the July August 2024 baseline survey of a quasi-experimental cluster study as part of the "Sepone MCH Grassroot Project" impact assessment (Protocol ID: 24-2329-00-00-00 on https://clinicaltrials.gov). Through community-based intervention by VHVs/VHWs, the project aims to promote MCH services among ethnic minorities in the Sepone district, with the Vilabuly district as the control. Both districts are located in Savannakhet Province, approximately 600 km from Vientiane, the capital city of the Lao PDR [22]. As of 2018, Sepone and Vilabuly, respectively, reported population sizes of 72,011 and 43,084, fertility rates of 2.9 and 2.1, and poverty rates of 44.0% and 40.8% [23, 24]. Each district has a hospital, with 14 health centers located in Sepone and 12 in Vilabuly [23, 25]. In addition, both districts share common features, such as extensive mountainous areas and a prevalence of ethnic minorities [22, 26]. The study sites included 19 villages in Sepone and 18 villages from Vilabuly, where we explored the potential associations between trust in VHVs/VHWs and facility-based follow-up PNC use via preintervention data.

### Responses

Women were eligible to enroll in the survey if they were mothers aged 15–49 with a live birth in the past 12 months, resided in the selected villages, and consented to participate. Mothers whose last pregnancy outcome was stillbirth were excluded from the study. In addition, cases in which children met the age criteria but the mother had died were excluded.

Before the survey, village leaders, health center staff, and VHVs/VHWs helped establish a list of potential respondents, mothers who gave birth within the past 18 months, and their locations. During household visits, child age eligibility (age ≤ 12 months) and the presence of mothers were confirmed based on their reports or from the medical handbook of mothers and babies, also called the "pink book."

### Sample size

The sample size for this study was estimated via logistic regression (*n* > 10 m), where n is the smaller number of subjects with or without the outcome, and m is the number of potential covariates for the regression [27]. With eight potential covariates, the study would require a minimum "n" of 80 facility-based follow-up PNC users. However, the sample size was determined by the limited number of eligible participants available within the targeted villages. Therefore, we conducted an exhaustive sampling, enrolling 241 mothers: 110 from Sepone and 131 from Vilabuly. To maximize participation with a response rate of at least 90.0%, we conducted multiple household visits to reach mothers who were initially unavailable. While the sample size may be small, exhaustive sampling allows the inclusion of almost all eligible subjects and ensures that the data represent the target population [28].

### Data collection

Data were collected through household visits in 37 villages (Table 5) by trained medical graduates via structured questionnaires prepared in Epi Info in English and translated into the Lao language. The questionnaire was administered in common local languages with a translator as needed. The survey questions were closed-ended, with some allowing multiple choices and Likert scale grading. They aimed to assess respondents' socioeconomic and demographic characteristics, previous MCH service use, and trust indicators in VHVs/VHWs. Mothers' self-reported information on service utilization and information (age of mother, age of child, etc.) were validated using information recorded in the mothers’ pink books.

### Variables and measurement

#### Main outcome

The primary outcome variable of the study was mothers' use of follow-up PNC services in a health facility at any time after childbirth. Participants’ responses to the question on using facility-based follow-up PNC services were categorized as "yes" if they reported visiting a health facility for a PNC after being discharged from their place of delivery and "no" if they reported otherwise. We considered follow-up PNC use regardless of whether the mothers used immediate PNC provided within 2 h after delivery and before discharge. Moreover, our definition did not distinguish postpartum visits that were hospital readmissions and did not apply a time limit for the follow-up PNC visits, as the present study prioritizes addressing the low coverage of visits.

#### Predictor variables

Based on the relevant literature on the determinants of obstetric care use, several predictor variables were designed [11, 29, 30]. The variables were then used as collected, and others were recategorized or combined as composites using question items where necessary.

The primary predictor variable, "trust in VHVs/VHWs," was measured as a composite variable of five questions from mothers' perspectives on the capacity of VHVs/VHWs to provide emotional support, provide relevant information, dedicate sufficient discussion time, be perceived as skillful, and be likely to consult VHVs/VHWs for future pregnancy-related care [19]. Cronbach's alpha (0.823) indicated a strong internal consistency of the items, and factor analysis supported combining all the items into the single construct of "trust in VHV/VHW," which was standardized and categorized into three levels (low, moderate, and high) [31]. Other predictor variables used in logistic regression were the sociodemographic characteristics of the respondents and their households (age, wealth assets, educational attainment, head of household, husband's occupation, etc.), previous health service use (place of delivery, number of ANC visits, use of PNC services, etc.), and the level of adherence to traditional gender norms. The wealth tertile variable was constructed as equal-sized groups of low-, middle-, and high-wealth women based on ownership of eleven assets (electricity, motorcycles, livestock, agricultural land, etc.) using principal component analysis [32, 33]. Adherence to traditional gender norms was constructed to rank respondents' beliefs (strong, moderate, and high) regarding observed and injunctive norms through four items (i.e., women obeying husbands in all matters, women requiring husbands’ permission for access to medical services, women being responsible for children's care, and husbands as primary household earners) [34].

#### Data analysis

Data analysis was performed using Stata (ver. 17, Stata Corp). Descriptive analyses helped us understand the demographic characteristics of the study population. We used chi-square tests (Fisher’s exact tests where the expected cell count was < 5) to compare health service utilization among mothers in the Sepone and Vilabuly districts. Binary standard logistic regression was used to assess the associations between the primary outcome and predictor variables. Then, via backward stepwise analysis, we selected covariates that influenced the outcome for inclusion in the multivariate logistic regression analysis while retaining essential sociodemographic variables, such as age and wealth tertiles. Due to to the small number of clusters (two districts) and minimal numbers of respondents per household (only one household had two respondents) and village (many villages had fewer than five respondents), which may induce unreliable random effects measurements, multilevel analysis with these factors was not feasible [35–37]. The most reasonable approach was to group villages by health facility catchment area; these areas were used as cluster levels in multilevel binary logistic regression. Nevertheless, the intraclass correlation coefficient was near zero (1.64e-33), suggesting the removal of random effects. Also, the interaction effect between trust and district variables was tested but found to be non-significant and was omitted from the final regression model. Statistics are reported with 95% confidence intervals (95% CIs), and *p* values of < 0.05 were considered to indicate statistical significance.

## Results

### Sociodemographic and economic characteristics of the respondents

Table 1 shows the distributions of the respondents according to several sociodemographic characteristics in the Sepone and Vilabuly districts.

**Table 1:** Sociodemographic characteristics of the Sepone and Vilabuly respondents

	Sepone district			Vilabuly district			*p* values for chi 2
	*n*	%	95% CI	*n*	%	95% CI	
Participant age groups (*N* = 241)							0.427^α^
≤ 19 years	22	20.0	13.5–28.6	28	21.4	15.2–29.3	
20–24 years	47	42.7	33.8–52.2	45	34.4	26.7–42.9	
25–29 years	15	13.6	8.4–21.4	31	23.7	17.1–31.7	
30–34 years	18	16.4	10.5–24.5	19	14.5	9.4–21.7	
35–39 years	7	6.4	3.1–12.8	7	5.3	2.6–10.8	
≥40 years	1	0.9	0.1–6.2	1	0.8	0.1–5.3	
Educational attainment of respondents (*N* = 241)							0.009^α^
No education	55	50.0	40.7–59.3	49	37.4	29.5–46.0	
Elementary school	25	22.7	15.8–31.5	25	19.1	13.2–26.8	
Middle school	23	20.9	14.3–29.6	31	23.7	17.1–31.8	
High school	5	4.6	1.9–10.5	24	18.3	12.6–25.9	
University or vocational school	2	1.8	0.5–7.0	2	1.5	0.4–5.9	
Respondent's marital status (*N* = 241)							0.459 ^α^
Currently married or living together	108	98.2	93.0–99.5	126	96.2	91.1–98.4	
Divorced/Widowed	2	1.8	0.5–7.0	5	3.8	1.6–8.9	
Parity groups (*N* = 241)							0.658
One	36	32.7	24.6–42.1	44	33.6	26.0–42.1	
Two	34	30.9	23.0–40.2	38	29	21.9–37.4	
Three	16	14.6	9–1–22.5	26	19.9	13.9–27.6	
Four or more	24	21.8	15.0–30.5	23	17.6	11.9–25.1	
Age of last child (*N* = 241)							0.010
0–4 months	35	31.8	23.8–41.1	57	43.5	35.2–52.2	
5–8 months	33	30.0	22.1–39.2	47	35.9	28.1–44.4	
9–12 months	42	38.2	29.6–47.6	27	20.6	14.5–28.4	
Ethnicity (*N* = 234)							< 0.001
Puthai/Lao Thai	31	28.4	20.7–37.7	47	37.6	29.5–46.4	
Mangol	23	21.1	14.4–29.8	68	54.4	45.6–63.0	
Tri	55	50.5	41.1–59.8	10	8.0	4.3–14.3	
Wealth tertiles (*N* = 240)							< 0.00
Poor	50	45.9	36.7–55.3	31	23.7	17.1–31.7	
Middle	35	32.1	24.0–41.5	44	33.6	26.0–42.1	
Rich	24	22.0	15.2–30.8	56	42.8	34.5–51.4	
Head of household (*N* = 241)							0.071 ^α^
Wife (participant)	1	0.9	0.1–6.2	0	0.0	–	
Husband	48	43.6	34.6–53.1	42	32.1	24.6–40.6	
Parent of wife	17	15.5	9.8–23.5	36	27.5	20.5–35.8	
Parent of husband	43	39.1	30.4–48.5	51	38.9	30.4–47.6	
Brother/sister or other	1	0.9	0.1–6.2	2	1.5	0.4–5.9	
Husband age groups (*N* = 241)							0.842
≤ 19 years	8	7.3	3.7–13.9	14	10.7	6.4–17.3	
20–24 years	33	30.0	22.1–39.2	34	26.0	19.1–34.2	
25–29 years	31	28.2	8.4–21.4	32	24.4	17.1–31.7	
30–34 years	15	13.6	8.4–21.4	23	17.6	11.9–25.1	
35–39 years	12	10.9	6.3–17.3	14	10.7	6.4–17.3	
≥ 40 years	11	10.0	5.6–17.2	14	10.7	6.4–17.3	
Husband's education (*N* = 228)							0.010 ^α^
No education	34	32.1	23.9–41.6	22	18.0	12.2–25.9	
Elementary school	31	29.3	21.3–38.6	29	23.8	17.0–32.2	
Middle school	24	22.6	15.6–31.6	31	25.4	18.4–33.9	
High school	16	15.1	9.4–23.3	32	26.2	19.2–34.8	
University or vocational school	1	0.9	0.1–6.5	8	6.6	3.3–12.6	
Husband's occupation (*N* = 234)							< 0.001 ^α^
No occupation	0	0.0	–	3	2.4	0.8–7.2	
Farmer	102	94.4	88.1–97.5	95	75.4	67.1–82.2	
Government employee	1	0.9	0.1–6.3	2	1.6	0.4–6.2	
Works in a private company	1	0.9	0.1–6.3	25	19.8	13.7–27.8	
Self–employed and other	4	3.7	1.4–9.5	1	0.8	0.1–5.5	

^α^Fisher test *p* value

Overall, the study surveyed 241 mothers, with a mean age of 24 years (SD: 5.7), of whom 110 resided in Sepone, where the majority of the respondents (50.5%) were of the Tri ethnicity, and 131 resided in Vilabuly, where the majority of the respondents (54.4%) were of the Mangol ethnicity. Almost all respondents, > 95%, reported being married or living with a male partner. Most respondents and their male partners were between 20 and 29 years of age, with most households in both districts headed by husbands or husbands' parents. Parity was similar across districts, with almost two-thirds of respondents reporting at least two children. The mean age of the children was 5.6 (SD: 3.3) months in Vilabuly District and 6.8 (SD: 3.7) months in Sepone District.

Educational attainment differed significantly across the two districts (*p* = 0.009): half of the respondents in Sepone had no formal education, and high school attainment was rare (4.6% in Sepone vs. 18.3% in Vilabuly). In Sepone, 32.1% of husbands had no formal education, compared to 18.0% in Vilabuly, where high school and university or vocational education levels were also higher. Moreover, husbands' occupations varied significantly across districts (*p* < 0.001). While the majority of husbands were farmers, this was more common in Sepone (94.4%) than in Vilabuly (75.4%). In Vilabuly, company employment was notable, with almost 20.0% of husbands working in a company. Sepone also had a greater proportion of respondents from poor households (45.9%) compared to 23.7% in Vilabuly, where over 40.0% of respondents fell into the rich tertile.

### Comparison of maternal health indicators, perceptions, and interactions with VHVs/VHWs among Sepone and Vilabuly district respondents

Table 2 compares the maternal and health indicators and women's engagement with VHVs/VHWs between Sepone and Vilabuly. While the results revealed significant differences in trust, satisfaction with VHVs/VHWs, access to healthcare, and adherence to traditional gender norms between the two districts, the use rates of ANC services, facility-based delivery, and PNC services were similar.

**Table 2:** Comparison of maternal and health indicators and women's engagement with VHVs/VHWs between Sepone and Vilabuly

	Sepone district			Vilabuly district			*p* values
	*n*	%	95% CI	*n*	%	95% CI	
Trust in VHVs/VHWs (*N* = 241)							< 0.001
Low	24	21.8	15.0–30.5	70	53.4	44.8–61.8	
Moderate	77	70.0	60.8–77.9	54	41.2	49.9–77.9	
High	9	8.2	4.3–15.0	7	5.3	2.6–10.8	
Knowledge of any VHV/VHW (*N* = 241)							< 0.001
No	10	9.1	4.9–16.1	60	45.8	37.4–54.4	
Yes	100	90.9	83.4–95.1	71	54.2	45.6–62.6	
Having any contact with VHVs/VHWs (*N* = 234)							< 0.001
Never	18	16.7	10.7–25.0	83	65.9	57.0–73.6	
Either before or after delivery	41	38.0	29.3–47.5	16	12.7	7.9–19.8	
Both during and after pregnancy	49	45.4	36.2–54.9	27	21.4	15.1–29.5	
Adherence to traditional gender norms (*N* = 241)							< 0.001
Low adherence	27	24.6	17.4–33.5	49	37.4	29.5–46.0	
Moderate adherence	47	42.7	33.8–52.2	72	55.0	46.3–63.3	
High adherence	36	32.7	24.6–42.1	10	7.6	4.1–13.6	
Time to reach nearest health facility on foot (*N* = 239)							< 0.001
Within an hour	22	20.4	13.8–29.1	58	44.3	36.0–52.9	
An hour or more	86	79.6	70.9–86.2	73	55.7	47.1–64.0	
Road access to nearest health facility (*N* = 241)							< 0.001
Not hard to reach (flat or almost flat terrain)	42	38.2	29.6–47.6	83	63.4	54.7–71.2	
Hard to reach (hilly, river and other terrain types)	68	61.8	52.4–70.4	48	36.6	28.8–45.3	
Follow-up PNC in health facility (*N* = 237)							0.619
Yes	22	20.4	13.8–29.1	23	17.8	12.1–25.4	
No	86	79.6	70.9–86.2	106	82.2	74.6–87.9	
Number of ANC visits (*N* = 241)							0.552
No visit	14	12.7	7.7–20.4	15	11.5	7.0–18.2	
One to three visits	32	29.1	21.3–38.3	31	23.7	17.1–31.7	
Four or more visits	64	58.2	48.7–67.1	85	64.9	56.3–72.6	
Place of delivery (*N* = 241)							0.878
Health facility	84	76.4	67.5–83.4	102	77.9	69.9–84.2	
Home or other	26	23.6	16.6–32.5	29	22.1	15.8–30.1	
Delivery by cesarean section (*N* = 241)							0.935
Yes	7	6.4	3.1–12.8	8	6.1	3.1–11.8	
No	103	93.6	87.2–96.9	123	93.9	88.2–96.9	
PNC use within the first 2 h after delivery (*N* = 239)							0.641
Yes	78	72.2	63.0–79.9	91	69.5	61.0–76.8	
No	30	27.8	20.1–37.0	40	30.5	23.2–39.0	

In terms of trust in VHVs/VHWs, more than half (53.4%) of Vilabuly mothers had lower trust in VHVs/VHWs in comparison with mothers in Sepone, where 70.0% of mothers had at least moderate trust (*p* < 0.001). Similarly, knowledge of VHVs/VHWs significantly differed across districts. In Sepone, less than 10.0% of women did not know of any VHVs/VHWs, whereas in Vilabuly, this figure was five times greater: 45.8% (*p* < 0.001). Furthermore, 16.7% of mothers in Sepone had never interacted with VHVs/VHWs, whereas many more mothers in Vilabuly had never interacted with VHV/VHWs (65.9%). Over 40% of women from Sepone had contact with VHVs/VHWs during both the prenatal and postnatal periods.

Adherence to gender norms also varied significantly. Although most respondents had moderate adherence, more mothers in Sepone (32.7%) had high adherence to traditional gender norms compared to 7.6% in Vilabuly. Only 20.4% of women in Sepone reported that they could reach the nearest facility within an hour, compared to 44.3% in Valabuly. In addition, 61.8% of women in Sepone resided in hard-to-reach areas from the nearest health facility, compared to a lower proportion (36.6%) in Vilabuly.

Maternal health indicators were similar in the Sepone and Vilabuly districts. Facility-based follow-up PNC use was only 20.4% and 17.8% in Vilabuly (*p* = 0.619). In both districts, most women (over 58.0%) reported receiving at least four ANC visits, more than 76% delivered in a health facility, and approximately 70.0% received an immediate PNC (*p* > 0.05).

### Comparison of trust in VHVs/VHWs and access to healthcare between follow-up PNC users and nonusers

Table 3 highlights the differences between respondents who reported using follow-up PNC services in a health facility regarding the trustworthiness of VHVs/VHWs and the accessibility and use of other maternal health services. The results revealed significant differences in trust in VHVs/VHWs, immediate PNC use, number of ANC visits, road access, and walking time to the nearest health facility.

**Table 3:** Comparison of trust in VHVs/VHWs and accessibility to healthcare among follow-up PNC users and nonusers

Use of follow-up PNC in a health facility							
Variables	Yes			No			Chi^2^ *p* values
	*n*	%	95% CI	*n*	%	95% CI	
Trust in VHVs/VHWs (*N* = 237)							0.002
Low trust	12	26.6	15.8—41.4	80	41.7	34.9–48.8	
Moderate trust	25	55.6	41.9–69.3	104	54.1	47.0–61.2	
High trust	8	17.8	9.1–31.8	8	4.2	2.1–8.1	
Having any contact with VHVs/VHWs (*N* = 232)							0.008^α^
Never	17	37.8	24.9–52.7	84	44.9	37.9–52.1	
Either before or after delivery	5	11.1	4.7–24.1	50	26.7	20.9–33.8	
Both during and after pregnancy	23	51.1	36.7–65.3	53	28.3	22.3–35.3	
Immediate PNC (first 2 h after delivery) (*N* = 236)							< 0.001 ^α^
No	3	6.7	2.2–18.8	67	35.1	28.6–42.1	
Yes	42	93.3	81.2–97.8	124	64.9	57.9–71.4	
Time to the nearest health facility on foot (*N* = 235)							0.016
Within an hour	22	48.9	34.7–63.3	57	30	23.9–36.9	
An hour or more	23	51.1	36.8–65.3	133	70	63.1–76.1	
Road access to nearest health facility (*N* = 237)							0.005
Not hard to reach (flat or almost flat road)	32	71.1	56.3–82.5	92	47.9	40.9–55.0	
Hard to reach (hilly, river and other terrain)	13	28.9	17.5–43.7	100	52.1	45.0–59.1	
Place of delivery (*N* = 237)							0.003 ^α^
Health facility	42	93.3	81.2–97.8	140	72.9	66.2–78.8	
Home or other	3	6.7	2.2–18.8	52	27.1	21.2–33.8	
Number of ANC visits (*N* = 237)							0.008 ^α^
No ANC visit	2	4.4	1.1–16.2	27	14.1	9.8–19.8	
1–3 ANC visits	6	13.3	6.1–26.7	55	28.7	22.7–35.5	
≥4 ANC visits	37	82.2	68.2–90.9	110	57.3	50.2–64.1	

^α^Fisher’s test *p* value

High trust in VHVs/VHWs was more than fourfold greater among follow-up PNC users (17.8%) than among nonusers (4.2%). Almost all follow-up PNC users (93.3%) and 64.9% of nonusers reported having had an immediate PNC visit at the delivery place within the first 2 h after childbirth (p < 0.001), although 72.9% of the latter group delivered in a health facility. Similarly, a significantly higher percentage of follow-up PNC users (82.2%) had at least four ANC visits in comparison with nonusers (57.3%) (p = 0.008). PNC users also reported also having more frequent interactions with VHVs/VHWs compared to women who did not use follow-up PNC. Furthermore, regarding accessibility of health facilities, a significantly greater proportion of PNC users (71.1%) resided in areas that were not too hard to reach (i.e., flat or almost flat terrain) in comparison with nonusers (47.9%), whereas nonusers of follow-up PNC resided at an hour or more away from the nearest health facility.

### Trust in VHVs/VHWs and other facility-based follow-up PNC use predictors

The bivariate analysis in Table 4 revealed that the level of trust in VHVs/VHWs, adherence to traditional gender norms, and the time required to reach the nearest health facility on foot and having immediate PNC were associated with the use of facility-based follow-up PNC. Compared with mothers with low trust, those with high trust in VHVs/VHWs were 6.67 times more likely to use follow-up PNC (*p* < 0.001). Moderate trust showed a weaker positive association; however, the difference was not statistically significant (COR: 1.6; *p* > 0.05). In addition, women who reported needing more than 1 h to access a health facility were less likely to attend follow-up PNC (COR: 0.45; p < 0.05). Having older children (9–12 months) was associated with more than twice the odds of follow-up PNC use among respondents with young children (0–4 months) (COR: 2.34; *p* < 0.05). Immediate PNC within the first 2 h after delivery was also strongly associated with follow-up PNC use (COR: 7.56; *p* < 0.001). Increased adherence to traditional gender norms reduced the odds of follow-up PNC use, with the lowest effect observed among women with high adherence (COR: 0.11; *p* < 0.01).

**Table 4:** Bivariate and multivariate logistic regression analyses of the associations between trust in VHVs/VHWs and follow-up PNC use across different models

Variable	Crude OR	95% CI	Model 1		Model 2	
			Adjusted OR	95% CI	Adjusted OR	95% CI
Trust in VHVs/VHWs						
Low trust	Ref.					
Moderate trust	1.6	0.8–3.4	2.13	0.9–4.9	2.72	1.0–7.6
High trust	6.67***	2.1–21.1	10.74***	2.9–39.8	12.25**	2.2–67.8
District						
Vilabuly district	Ref.					
Sepone district	1.18	0.6–2.3	1.15	0.5–2.5	1.76	0.7–4.6
Participant age groups						
≤19 years	Ref.					
20–24 years	2.04	0.8–5.5	2.3	0.8–6.8	2.69	0.8–8.7
≥25 years	1.83	0.7–4.9	2.21	0.7–6.6	2.37	0.7–7.6
Educational attainment of respondent						
No formal education	Ref.					
Elementary school	1.21	0.5–3.0	1.15	0.4–3.1	0.73	0.2–2.2
Middle/High school/University and Vocational	1.63	0.8–3.4	1.33	0.6–3.2	0.87	0.3–2.3
Age of last child						
0–4 months	Ref.					
5–8 months	1.15	0.5–2.7	1.2	0.5–3.0	1.15	0.4–3.1
9–12 months	2.34*	1.1–5.2	3.20**	1.3–7.9	3.16*	1.2–8.5
Adherence to traditional gender norms						
Low adherence	Ref.					
Moderate adherence	0.48*	0.2–1.0	0.42	0.2–0.9	0.39	0.2–0.9
High adherence	0.11**	0.0–0.5	0.08**	0.0–0.4	0.07**	0.0–0.4
Accessibility and health service utilization factors						
Having any contact with VHVs/VHWs						
Never	Ref.		–	–	Ref.	
Either before or after delivery	0.49	0.2–1.4	–	–	0.23*	0.1–0.9
Both during and after pregnancy	2.14	1.0–4.4	–	–	0.76	0.2–2.4
Time to the nearest health facility on foot						
Within an hour	Ref.		–	–	Ref.	
An hour or more	0.45*	0.2–0.9	–	–	0.40*	0.2–0.9
Immediate PNC (first 2 h after delivery)			–	–		
No	Ref		–	–	Ref.	
Yes	7.56***	2.3–25.3	–	–	6.80**	1.8–26.0

*p value < 0.05; **p value < 0.01; *** *p* value < 0.001

*OR* odds ratio, *CI* confidence interval, *Ref* reference group

After adjusting for other covariates, the effect of high trust in VHVs/VHWs on follow-up PNC remained strong across the models, reaching an odds ratio of 12.25 in model 2 (*p* < 0.01). Similarly, moderate trust was positively associated with follow-up PNC but was not statistically significant. Other factors associated with follow-up PNC use included having a 9–12-month-old child (AOR model 2: 3.16%; *p* < 0.05) and using immediate PNC (AOR model 2: 6.80; *p* < 0.001), for which effects also remained consistent throughout the adjusted analyses.

Conversely, mothers living an hour or more from a health facility, those who had contact with VHVs/VHWs during the pre- and postnatal periods, and those with moderate to high adherence to traditional gender norms were less likely to use follow-up PNC than their counterparts. Moreover, factors such as the district of residence, participant age group, and educational attainment did not show statistically significant associations with facility-based follow-up PNC use.

## Discussion

The present study revealed very low uptake (19.0%) but similar levels of facility-based follow-up PNC in Sepone and Vilabuly, despite differences in access to healthcare and engagement with VHVs/VHWs; low trust in VHVs/VHWs was more prevalent among follow-up PNC nonusers (26.6%) than among users (41.7%); high trust was associated with favorable odds of using follow-up PNC. In addition, having an older child (9–12 months) and immediate PNC increased the odds of follow-up PNC use. Surprisingly, having contact with VHVs/VHWs during the pre- or postnatal periods reduces the likelihood of follow-up PNC. Moreover, greater adherence to traditional gender norms or remoteness from a health facility were barriers to follow-up PNC use.

The notably low follow-up PNC use in the present study aligns with previous evidence of the substantial lack of initiation and continuation of PNC services in resource-limited settings [38, 39]. Similar minimal rates (3.7% in 2017) of subsequent PNC visits after discharge from the delivery location were reported in Lao PDR despite the implementation of the free MCH service access policy in 2012 [10, 11, 40]. Several studies have emphasized the need to understand and address barriers related to cultural beliefs, awareness of PNC benefits, and accessibility factors [40, 41]. For example, in Indonesia, Uganda, and Zambia, studies have shown that women do not attend postnatal services due to perceived low value or need, particularly in the absence of childbirth-related complications [41–43].

Despite differences in access and VHVs/VHWs engagement, Sepone and Vilabuly had similar maternal health service coverage, highlighting district-specific challenges to address. In Sepone, where mothers had greater trust in VHVs/VHWs and frequent interactions, the number of ANC visits and facility-based deliveries were relatively high despite barriers to facility access. The findings suggest that VHVs/VHWs may provide essential support in promoting service utilization, as reported in a previous study [44]. Moreover, the lack of follow-up PNC (20.4%) in the district provides an opportunity to leverage the established importance of VHVs/VHWs for better advocacy for follow-up PNC services while navigating logistical barriers. In contrast, in Vilabuly, where mothers had less engagement with VHVs/VHWs, the easier access to health facilities might have contributed to maternal health service coverage levels similar to those in Sepone. While considering local challenges in Vilabuly, enhancing the role and visibility of VHVs/VHWs and mothers' reliance on them may further optimize service uptakes.

Trust in VHVs/VHWs differed significantly among facility-based follow-up PNC users and nonusers, with high trust associated with a substantial increase in the odds of follow-up PNC use (AOR model 2: 12.25). These findings align with the relevant literature, which suggests that trust in CHWs, such as VHVs, can promote health-seeking behaviors, including formal healthcare services [21, 45, 46]. According to Darden and Macis (2024), trust—in addition to income and education—is well-known to play a primary role in healthcare utilization, even when communities have limited knowledge about the importance of the service [45]. Trust helps strengthen the relationship between health workers and mothers, making it easier for mothers to seek advice and services. The varying levels of trust in VHVs/VHWs among women also suggest that service users and nonusers may have different exposure to VHVs/VHWs activities, subsequently influencing their views [19, 46, 47]. This is reflected in our findings, where the rate of follow-up PNC use was the greatest among those who reported meeting VHVs/VHWs both during and after pregnancy (51.1%).

Unexpectedly, after adjustment for covariates, women who had contact with VHVs/VHWs were less likely to utilize facility-based follow-up PNC services than those who had no contact. While this finding challenges the assumption that increased interactions with healthcare professionals would naturally improve service utilization, it raises the question of the extent to which mothers perceived their needs to be sufficiently met by VHVs/VHWs. Despite their essential role, VHVs/VHWs might be perceived as adequate substitutes for formal healthcare, reducing the perceived need for the latter [19, 48]. While regular interactions between VHVs/VHWs and mothers are beneficial, the quality of those contacts is equally important. Actions for improvement must integrate VHVs/VHWs's efforts with formal healthcare systems with constant support and supervision [49–51]. VHVs/VHWs should emphasize the complementary roles of community-based care and health facility services in communities. Likewise, further qualitative studies on mothers’ perceived value of community-based and facility-based care are essential.

Mothers of older children (9–12 months) were more likely to seek follow-up PNC at health facilities, which deviates from the WHO's recommended four PNC visits in the first six postpartum weeks for increased survival chances for mothers and newborns [4]. The delay in follow-up PNC uptake suggests that over time, mothers may become familiar with health systems, become more aware of the benefits of health services, or become more concerned about child development, motivating positive health-seeking behavior [52]. However, the lack of PNC use among mothers with younger infants constitutes missed opportunities for timely intervention for the most vulnerable ages to neonatal complications [4]. In addition, immediate PNC within the first 2 h after delivery greatly influences follow-up PNC use, reinforcing the benefits of early contact with health professionals in establishing ongoing healthcare practices [53–55]. As highlighted by previous studies, addressing geographic accessibility barriers and restrictive norms to women's autonomy in health decisions is essential in follow-up PNC use [56, 57]. Effective interventions to transform gender norms may require engaging men and educating women [58–60].

## Limitations

The present study had several limitations. First, due to the study design, we could not infer causality between factors; however, we found potential associations for further investigation. Second, despite validation efforts with pink books, we relied primarily on self-reported information from mothers. Third, not distinguishing postpartum visits that might have been hospital readmissions could have inflated the proportion of facility-based follow-up PNC. Nonetheless, this could further highlight the already low coverage of follow-up PNC which requires attention.

## Conclusion

The study highlights the lack of facility-based follow-up PNC use in rural Lao PDR and the critical role of women's trust in VHVs/VHWs in potentially fostering improvement. While ensuring regular quality contact with mothers, VHVs/VHWs may emphasize the complementary benefit of formal healthcare services [61, 62]. In addition to improving mothers' reliance on VHVs/VHWs, future interventions may promote immediate PNC, address physical accessibility challenges, and address restrictive gender norms through male involvement. Furthermore, qualitative studies may help deepen the understanding of women's beliefs about follow-up PNC to design appropriate educational activities.

## Figures and Tables

**Fig. 1:**
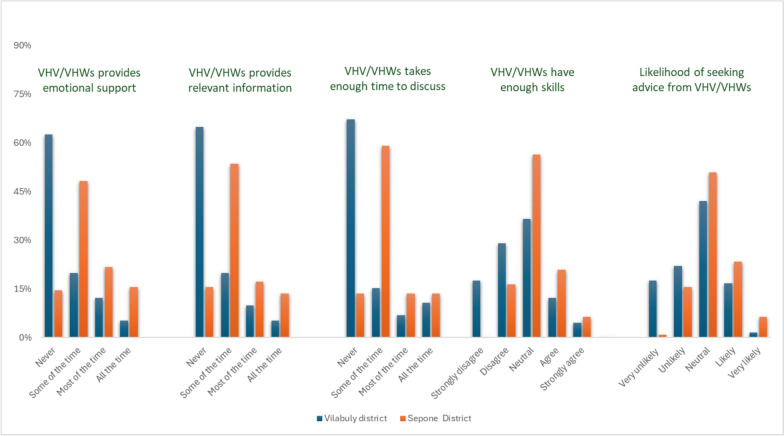
Percentages of responses to question items on trust in VHVs/VHWs

## Data Availability

No datasets were generated or analysed during the current study.

## Appendix

See Fig. 1 and Table 5

**Table 5:** Number of respondents by village

Sepone district		Vilabuly district	
Village names	Number of respondents	Village names	Number of respondents
Arlainoy	2	Dongyang	6
Dongnoy	7	Huaydarng	2
Huaycheangkao	7	Huaysuan	5
Kadub	9	Keangleknuea	7
Kaloukkao and Kaloukmai	8	Keanglektai	9
Keangkiew	4	Kokmark	8
Keangluangnoy	6	Lardeangnoy	3
Keangpeanai	3	Nabalu	4
Keangthongnok	5	Nater	6
Kheaving	3	Numkheep	9
LaAorkao	5	Numlap	3
Mueangchannok	3	Nummee	9
Munchy	9	Padong	13
Panga	9	Phaphilarng	10
Poung	6	Poungpor	13
Sadueng	6	Thangbeangkeangluang	7
Sawed	3	That	6
Vangmorthoum	11	Torhuea	11
Vangyeangnoy	4		
Total	110	Total	131

## Abbreviations

ANC: Antenatal care
CHW: Community health worker
FGD: Focus group discussion
JICA: Japan International Cooperation Agency
MCH: Maternal and child health
NECHR: National Ethics Committee for Health Research
PNC: Postnatal care
VHV/VHW: Village health volunteer or worker

## References

[1] World Health Organization, “Newborn mortality.” https://www.who.int/news-room/fact-sheets/detail/newborn-mortality. Accessed 05 Jan 2025.

[2] World Health Organization, Postnatal care of the mother and newborn 2013, *World Health Organization*, pp. 1–72, 2013, [Online]. http://apps.who.int/iris/bitstream/10665/97603/1/9789241506649_eng.pdf

[3] Center for Disease Control, Pregnancy-Related Deaths: Data From Maternal Mortality Review Committees in 38 U.S. States, 2020 | Maternal Mortality Prevention | CDC. [Online]. https://www.cdc.gov/maternal-mortality/php/data-research/index.html. Accessed 07 Jan 2025.

[4] World Health Organization, WHO recommendations on maternal and newborn care for a positive postnatal experience, World Health Organization, p. 224, 2022, [Online]. https://www.who.int/publications/i/item/9789240045989 35467813

[5] Langlois ÉV, Miszkurka M, Zunzunegui MV, Ghaffar A, Ziegler D, Karp I. Systematic reviews Inequities in postnatal care in low-and middle-income countries: a systematic review and meta-analysis. Bull World Health Organ. 2015;93:259–70. 10.2471/BLT.14.140996. 26229190 10.2471/BLT.14.140996 PMC4431556

[6] Wilson EB, Niehaus L, Jiwani SS, Hazel EA, Maïga A, Amouzou A. Delivery and postnatal care among women in 71 low- and middle-income countries: analyzing coverage gaps using household surveys. BMC Pregnancy Childbirth. 2024. 10.1186/s12884-024-06681-y. 39060978 10.1186/s12884-024-06681-y PMC11282627

[7] Ministry of Health and Lao Statistics Bureau, Lao Social Indicator Survey 2011–12 Final report, no. December. Vientiane, Lao PDR, 2012. 10.4135/9781412976961.n199.

[8] Lao Statistics Bureau, Key Indicators Report Lao Social Indicator Survey III-2023, 2024.

[9] Lao Statistics Bureau, Lao Social Indicator Survey II 2017, Survey Findings Report. Vientiane, Lao PDR, 2018.

[10] Ahissou NCA, Nonaka D, Takeuchi R, de los Reyes C, and ..., “Trend of sociodemographic and economic inequalities in the use of maternal health services in Lao People’s Democratic Republic from 2006 to 2017: MICS …,” Trop Med Health, 2023, 10.1186/s41182-023-00548-2. 10.1186/s41182-023-00548-2 PMC10585846 37858190

[11] Quayle T, factors associated with immediate postnatal care in Lao People’s Democratic Republic: an analysis of the 2017 Lao People’s Democratic Republic multiple indicator cluster survey, UNLV Theses, Dissertations, Professional Papers, and Capstones. 4613., Dec. 2022, 10.34917/35777496. 10.1186/s12884-026-08899-4 PMC13088657 41814214

[12] Lao Ministry of Health, Primary Healthcare Policy, 2019.

[13] Lao Ministry of Health, " [Recommendations regarding Village Health Volunteers (VHV)]", 2020.

[14] Nonaka D, et al. Primary health care situations in remote rural villages of the Savannakhet province, Lao People’s Democratic Republic. Trop Med Health. 2022. 10.1186/s41182-022-00482-9. 36443857 10.1186/s41182-022-00482-9 PMC9703750

[15] Pongvongsa T, Nonaka D, Kobayashi J, Mizoue T, Phongmany P, Moji K. Determinants of monthly reporting by village health volunteers in a poor rural district of Lao PDR. Southeast Asian J Trop Med Public Health. 2011;42(5):1269–81. 22299454

[16] Le Roux KW, et al. Community health workers impact on maternal and child health outcomes in rural South Africa - a non-randomized two-group comparison study. BMC Public Health. 2020;20(1):1–14. 10.1186/S12889-020-09468-W/FIGURES/3. 32943043 10.1186/s12889-020-09468-w PMC7496216

[17] Gilmore B, McAuliffe E. Effectiveness of community health workers delivering preventive interventions for maternal and child health in low- and middle-income countries: a systematic review. BMC Public Health. 2013;13(1):1–14. 10.1186/1471-2458-13-847/TABLES/2. 24034792 10.1186/1471-2458-13-847 PMC3848754

[18] Van Iseghem T, et al. The role of community health workers in primary healthcare in the WHO-EU region: a scoping review. Int J Equity Health. 2023;22(1):1–15. 10.1186/S12939-023-01944-0/TABLES/3. 37474937 10.1186/s12939-023-01944-0 PMC10357780

[19] Sripad P, Mcclair TL, Casseus A, Hossain S, Abuya T, Gottert A. Measuring client trust in community health workers: a multi-country validation study. J Glob Health. 2021;11:7009. 10.7189/jogh.11.07009. 10.7189/jogh.11.07009 PMC7956104 33763223

[20] Ahmed S, et al. Community health workers and health equity in low- and middle-income countries: systematic review and recommendations for policy and practice. Int J Equity Health. 2022;21(1):1–30. 10.1186/S12939-021-01615-Y. 35410258 10.1186/s12939-021-01615-y PMC8996551

[21] Watkins AA. Community health workers’ efforts to build health system trust in marginalised communities: a qualitative study from South Africa. BMJ Open. 2021;11:44065. 10.1136/bmjopen-2020-044065. 10.1136/bmjopen-2020-044065 PMC8137175 34011590

[22] Lao Statistics Bureau, " (National Statistics Center, Ministry of Planning and Investment, Annual Statistics Book 2022)", 2022.

[23] Lao Statistics Bureau, Where are the poor? Lao PDR 2015 Census-based poverty map’ Province and District Level Results, 2016.

[24] Lao Statistics Bureau, Ministry of Planning and Investment District Population Projections, Vientiane, 2019.

[25] Savannakhet Health Provincial Office, 2023 " (Summary of information about small hospitals under Kaysone Phomvihane City and 14 districts Including the distances from", 2023.

[26] Xiao C, Li P, Feng Z, You Z, Jiang L, Boudmyxay K. Is the phenology-based algorithm for mapping deciduous rubber plantations applicable in an emerging region of northern Laos? Adv Space Res. 2020;65(1):446–57. 10.1016/j.asr.2019.09.022.

[27] Peduzzi P, Concato J, Kemper E, Holford TR, Feinstein2~3~4, A simulation study of the number of events per variable in logistic regression analysis, 1996. 10.1016/s0895-4356(96)00236-3 8970487

[28] Sarker M, Al-Muaalemi MA, Sampling Techniques for Quantitative Research, Principles of Social Research Methodology, pp. 221–234, 2022, 10.1007/978-981-19-5441-2_15.

[29] Dada S, Tunçalp Ö, Portela A, Barreix M, Gilmore B. Community mobilization to strengthen support for appropriate and timely use of antenatal and postnatal care: A review of reviews. J Glob Health. 2021. 10.7189/jogh.11.04076. 35003714 10.7189/jogh.11.04076 PMC8710228

[30] Herwansyah H, Czabanowska K, Kalaitzi S, Schröder-Bäck P. The utilization of maternal health services at primary healthcare setting in Southeast Asian Countries: a systematic review of the literature. Sexual Reprod Healthc. 2022. 10.1016/j.srhc.2022.100726. 10.1016/j.srhc.2022.100726 35462125

[31] Taber KS. The use of cronbach’s alpha when developing and reporting research instruments in science education. Res Sci Educ. 2018. 10.1007/s11165-016-9602-2.

[32] Filmer D, Pritchett LH. Estimating wealth effects estimating wealth effects without expenditure data—or tears: an application to educational enrollments in States of India * Deon Filmer and Lant H. Pritchett. Demography. 2001;38(1):115–32. 11227840 10.1353/dem.2001.0003

[33] Ahissou NCA, et al. Modern contraceptive use among adolescent girls and young women in Benin: a mixed-methods study. BMJ Open. 2022;12(1):1–12. 10.1136/bmjopen-2021-054188. 10.1136/bmjopen-2021-054188 PMC8728422 34983766

[34] Sedlander E, Bingenheimer JB, Long MW, Swain M, Rimal RN. The G-NORM scale: development and validation of a theory-based gender norms scale. Sex Roles. 2022;87(5–6):350–63. 10.1007/s11199-022-01319-9. 36168556 10.1007/s11199-022-01319-9 PMC9508194

[35] Snijders TAB, Bosker R, Multilevel analysis: an introduction to basic and advanced multilevel modeling, 1999. [Online]. https://www.researchgate.net/publication/44827177

[36] Schunck R, Cluster size and aggregated level 2 variables in multilevel models. A cautionary note, 2016;10(1):2016. 10.12758/mda.2016.005.

[37] MenceLeyrat C, Morgan KE, Leurent B, Kahan BC. Education Corner Cluster randomized trials with a small number of clusters: which analyses should be used? Int J Epidemiol. 2018. 10.1093/ije/dyx169. 10.1093/ije/dyy057 29596636

[38] Muriuki A, Yahner M, Kiragu M, De Graft-Johnson J, Izulla P, Nairobi K. On the road to universal coverage of postnatal care: considerations for a targeted postnatal care approach for at-risk mother-baby dyads in low-income and middle-income countries informed by a consultation with global experts Save the Children. BMJ Open. 2022;12:58408. 10.1136/bmjopen-2021-058408. 10.1136/bmjopen-2021-058408 PMC9198691 35701048

[39] Serbanescu F, et al. Individual, community, and health facility predictors of postnatal care utilization in rural tanzania: a multilevel analysis. Glob Health Sci Pract. 2023. 10.9745/GHSP-D-22-00502/-/DCSUPPLEMENTAL. 37640485 10.9745/GHSP-D-22-00502 PMC10461704

[40] Sato C, et al. Factors influencing the choice of facility-based delivery in the ethnic minority villages of Lao PDR: a qualitative case study. Trop Med Health. 2019;47(1):1–11. 10.1186/S41182-019-0177-2/FIGURES/1. 31516363 10.1186/s41182-019-0177-2 PMC6729018

[41] Sacks E, et al. Postnatal care experiences and barriers to care utilization for home- and facility-delivered newborns in Uganda and Zambia. Matern Child Health J. 2017;21(3):599–606. 10.1007/S10995-016-2144-4. 27475823 10.1007/s10995-016-2144-4

[42] Titaley CR, Hunter CL, Heywood P, Dibley MJ. Why don’t some women attend antenatal and postnatal care services? A qualitative study of community members’ perspectives in Garut, Sukabumi and Ciamis districts of West Java Province, Indonesia. BMC Pregnancy Childbirth. 2010;10(1):1–12. 10.1186/1471-2393-10-61/FIGURES/2. 20937146 10.1186/1471-2393-10-61 PMC2964562

[43] Titaley CR, Dibley MJ, Roberts CL. Factors associated with non-utilisation of postnatal care services in Indonesia. J Epidemiol Community Health. 2009;63(10):827–31. 10.1136/JECH.2008.081604. 19414443 10.1136/jech.2008.081604

[44] Toyama N, et al. Impact of village health volunteer support on postnatal depressive symptoms in the remote rural areas of Lao People’s Democratic Republic: a cross-sectional study. Trop Med Health. 2021. 10.1186/s41182-021-00316-0. 33785051 10.1186/s41182-021-00316-0 PMC8010948

[45] Darden ME, Macis M, Trust and Health Care-Seeking Behavior, 1050 Massachusetts Avenue Cambridge, MA 02138, May 2024. [Online]. https://www.socialscienceregistry.org/trials/11214

[46] Capotescu C, et al. Community health workers’ critical role in trust building between the medical system and communities of color. Am J Managed Care. 2022;28(10):497–9. 10.37765/AJMC.2022.89247. 10.37765/ajmc.2022.89247 36252168

[47] Albrecht S, Travaglione A. Trust in public-sector senior management. Int J Hum Resour Manag. 2003;14(1):76–92. 10.1080/09585190210158529.

[48] Breakthrough Action, Community Health Worker Literature Review: Demand Generation, 2023.

[49] Nida S, et al. A systematic review of the types, workload, and supervision mechanism of community health workers: lessons learned for Indonesia. BMC Primary Care. 2024. 10.1186/s12875-024-02319-2. 38468218 10.1186/s12875-024-02319-2 PMC10926673

[50] Kowitt SD, Emmerling D, Fisher EB, Tanasugarn C. Community health workers as agents of health promotion: analyzing Thailand’s village health volunteer program. J Community Health. 2015;40(4):780–8. 10.1007/s10900-015-9999-y. 25744815 10.1007/s10900-015-9999-y

[51] Nonaka D, et al. Successful mobile phone network-based approach to integration of the health care system in rural Laos: strengthening lay health worker performance. RRH. 2014. 10.22605/RRH2588. 24547711

[52] Nuwabaine L, et al. Factors associated with quality of postnatal care in Kenya: an analysis of the 2022 Kenya demographic and health survey. Arch Public Health. 2024;82:1. 10.1186/S13690-024-01433-Y. 39605079 10.1186/s13690-024-01433-y PMC11600649

[53] Khatri R, et al. Continuity and care coordination of primary health care: a scoping review. BMC Health Serv Res. 2023. 10.1186/s12913-023-09718-8. 37443006 10.1186/s12913-023-09718-8 PMC10339603

[54] Wu S, Du S, Feng R, Liu W, Ye W. Behavioral deviations: healthcare-seeking behavior of chronic disease patients with intention to visit primary health care institutions. BMC Health Serv Res. 2023. 10.1186/s12913-023-09528-y. 37189156 10.1186/s12913-023-09528-y PMC10185376

[55] Bekele SB, Yirdaw BW, Abuhay M, Gebremichael MA. Immediate postnatal care satisfaction and associated factors among postnatal women in public health facilities at Debre Markos Town, Northwest Ethiopia, 2021. Patient Prefer Adherence. 2022;16:137–47. 10.2147/PPA.S348597. 35082489 10.2147/PPA.S348597 PMC8784914

[56] Dotse-Gborgbortsi W, et al. Distance is ‘a big problem’: a geographic analysis of reported and modelled proximity to maternal health services in Ghana. BMC Pregnancy Childbirth. 2022;22(1):1–12. 10.1186/S12884-022-04998-0/FIGURES/5. 36045351 10.1186/s12884-022-04998-0 PMC9429654

[57] Okwaraji YB, Edmond KM. Proximity to health services and child survival in low- and middle-income countries: a systematic review and meta-analysis. BMJ Open. 2012. 10.1136/bmjopen-2012-001196. 22798257 10.1136/bmjopen-2012-001196 PMC3400076

[58] Betron Myra, “Addressing Gender Norms and Inequalities to Improve Maternal and Child Health: Describing and Building on the Evidence Base for Gender-Integrated Interventions – Maternal Health Task Force,” Havard T.H. Chan, School of Public Health. [Online]. https://www.mhtf.org/document/addressing-gender-norms-and-inequalities-to-improve-maternal-and-child-health-describing-and-building-on-the-evidence-base-for-gender-integrated-interventions/?form=MG0AV3. Accessed 15 Jan 2025.

[59] Lusambili AM, et al. Male involvement in reproductive and maternal and new child health: an evaluative qualitative study on facilitators and barriers from rural Kenya. Front Public Health. 2021;9: 644293. 10.3389/FPUBH.2021.644293/BIBTEX. 33968883 10.3389/fpubh.2021.644293 PMC8096930

[60] Gopal P, Fisher D, Seruwagi G, Taddese HB. Male involvement in reproductive, maternal, newborn, and child health: Evaluating gaps between policy and practice in Uganda. Reprod Health. 2020;17(1):1–9. 10.1186/S12978-020-00961-4/TABLES/1. 32718357 10.1186/s12978-020-00961-4 PMC7385888

[61] Sharma N, Harris E, Lloyd J, Mistry SK, Harris M. Community health workers involvement in preventative care in primary healthcare: a systematic scoping review. BMJ Open. 2019;9(12): e031666. 10.1136/BMJOPEN-2019-031666. 31852698 10.1136/bmjopen-2019-031666 PMC6937114

[62] Perry HB, et al. Community health workers at the dawn of a new era: 11. CHWs leading the way to ‘Health for All.’ Health Res Policy Syst. 2021;19(3):1–21. 10.1186/S12961-021-00755-5/TABLES/2. 34641891 10.1186/s12961-021-00755-5 PMC8506098

